# Prevalence and Associated Factors of Cigarette Smoking among South African Adolescents and Young Adults: A Systematic Review and Meta-Analysis Protocol

**DOI:** 10.3390/mps6050085

**Published:** 2023-09-11

**Authors:** Mukhethwa Londani, Olanrewaju Oladimeji

**Affiliations:** 1 Department of Public Health, Walter Sisulu University, Eastern Cape, Mthatha 5117, South Africa; ooladimeji@wsu.ac.za; 2 Directorate of Research and Innovation, Tshwane University of Technology, Pretoria 0001, South Africa

**Keywords:** South Africa, adolescents, young adults, tobacco use, systematic review, meta-analysis

## Abstract

Tobacco use, particularly the initiation of smoking during adolescence and young adulthood, represents a significant public health concern in South Africa. The influence of socio-cultural factors, marketing strategies of the tobacco industry, and accessibility of tobacco products have all been implicated in this context. This systematic review and meta-analysis protocol aims to scrutinise the body of literature on this issue, providing a comprehensive understanding of the patterns and determinants of tobacco use among South African adolescents and young adults, with an eye towards informing more effective policy interventions. The available literature for studies on tobacco use will be systematically searched and reviewed. Five international scholarly databases, namely PubMed, MEDLINE, EMBASE, Global Health, and Scopus, will be searched. Peer-reviewed studies will be included if they are conducted in South Africa or South African provinces and if they include the prevalence of tobacco use among adolescents and young adults aged between 12 and 24 years. The results of such an analysis can guide future policy designs, enabling them to be more targeted and thus more effective. The findings can also have implications for shaping global tobacco control strategies, given the transferability of successful interventions across different populations and cultural contexts. This protocol has been registered in the PROSPERO database (ID: CRD42023428369).

## 1. Introduction

Tobacco use is one of the most significant public health concerns for both users and society and often begins early in adult life. Globally, tobacco use has been recognized as an important risk factor for non-communicable diseases and an increased level of mortality [1,2]. Despite international initiatives to reduce tobacco use, the World Health Organization (WHO) estimated tobacco as the cause of more than 8 million deaths annually [3]. Nonetheless, there are more than one billion people smoking every day, and more than 80% of those people are found in low- and middle-income countries (LMICs) [4,5,6]. Further, it has been estimated that about 80% of established adult smokers started smoking before the age of 18 years [7,8]. Based on data from the Centers for Disease Control and Prevention in United States, 8.2% and 23.9% of middle school students and high school students, respectively, reported smoking tobacco [9]. As such, other studies have shown a high prevalence of cigarette smoking among adolescents and young adults residing in developing countries, especially Sub-Saharan Africa [10,11,12].

South Africa, on the other hand, had a higher prevalence of tobacco smoking among all ethnic groups since 1976 [13,14,15], which was one of the highest smoking rates in the continent [16]. According to the South African Global Adult Tobacco Survey conducted in 2021, it was found that 12.7 million people aged 15 years and above were currently using tobacco, making up one-third of the adult population [17]. Also, tobacco smoking results in a large burden of preventable diseases and cases of premature death in South Africa. Statistics South Africa reported that tobacco use contributed to approximately 20% of the total deaths in 2017 [18]. Each year, approximately 83,957 people die from tobacco-related diseases [18] in South Africa, with tuberculosis as the leading cause of death, followed by hypertensive diseases, and chronic lower respiratory diseases [19]. This number is expected to rise to 250,000 in the next few years if new interventions and tobacco controls are not implemented [14]. In addition to risks posed by tobacco users, non-smokers are also at health risks related to exposure to second-hand smoke [20].

South Africa successfully developed a strong tobacco control legislation to reduce the prevalence of tobacco smoking in the late 1980s and early 1990s [21], and also adopted the WHO Framework Convention on Tobacco Control (WHO FCTC) in 2005 as a response to the globalisation of the tobacco epidemic, thus being obligated to pass comprehensive tobacco control policies [22,23]. However, despite public health efforts, the use of tobacco smoking keeps increasing, especially among adolescents and young adults [21], where the prevalence has increased from 16.5% in 2008 to 16.9% in 2011. Thus, there is a necessity for newer studies to emerge, allowing for a better understanding of how effective the restriction and enforcement of South African Tobacco Control policies are, as well as efforts taken to reduce tobacco smoking in South Africa.

Factors associated with tobacco use among young people include cultural and social status, parental smoking, psychological factors (e.g., gender and parental divorce) [24,25], and socializing with smokers [26]. Therefore, a better understanding of the epidemiology of tobacco smoking among adolescents and young adults is required to develop more effective tobacco-focused interventions in South Africa. The present study will address the abovementioned gaps by systematically reviewing published articles on tobacco use among adolescents and young adults to estimate the prevalence of tobacco use and to inform targeted intervention efforts aimed at reducing the level of smoking and associated harms in South Africa.

The objective of this systematic review and meta-analysis is to estimate the pooled prevalence of tobacco use among South African adolescents and young adults, and to assess the association between current tobacco use and socio-demographic variables (such as gender, race, household income, and education level). Current tobacco use will be defined as smoking once or more than once every day or every other day within 30 days. This study will include adolescents and young adult smokers who are school-going and school dropouts.

## 2. Materials and Methods

### 2.1. Overall Approach

**Figure 1:**
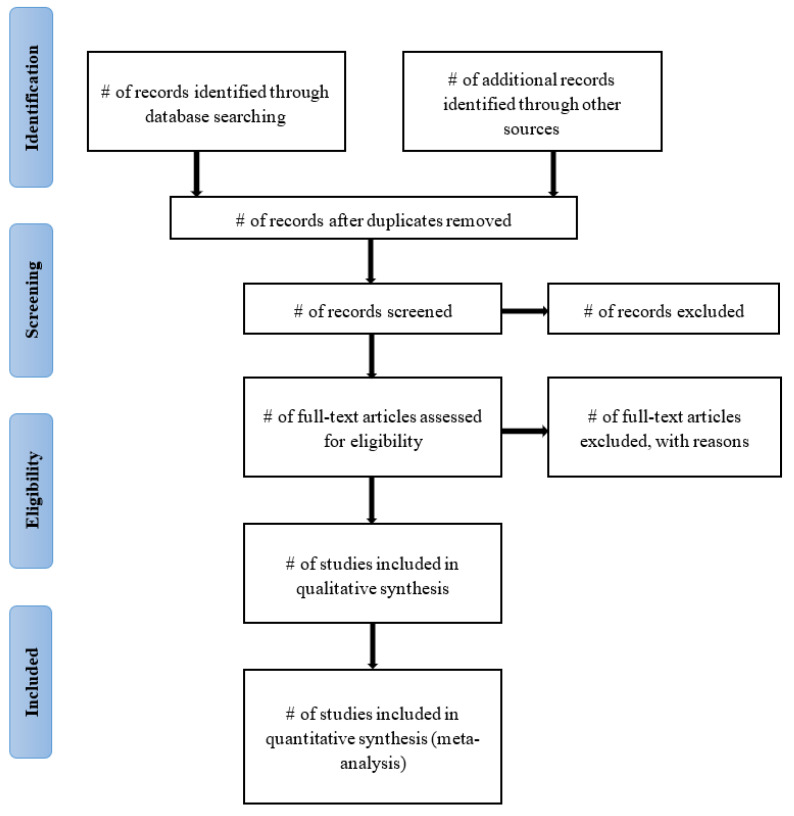
PRISMA flow-chart of systematic search process.

A systematic review will be performed using the Preferred Reporting Items for Systematic Reviews and Meta-Analysis (PRISMA) guidelines [27] on studies concerning tobacco use among adolescents and young adults in South Africa. This approach will be followed to describe the studies’ identification, selection, and inclusion in the review as shown in Figure 1. If there are any amendments to this protocol, the changes will be documented and the date of the amendment will be included. 

### 2.2. Population of Interest

The population of interest includes South African adolescents and young adults who have smoked tobacco in the last 30 days and who are aged between 12 and 24 years.

### 2.3. Search Strategy

Relevant South African articles, published in the English language, will be identified by searching the five international scholarly databases, namely PubMed, MEDLINE, EMBASE, Global Health, and Scopus. Google Scholar will also be searched to increase the chances of finding more relevant articles related to tobacco smoking. To successfully retrieve relevant articles, the following key terms will be used during the search: (tobacco use OR tobacco consumption OR cigarette smoking OR smoking) AND (adolescents OR young people OR school learners OR young adults OR youth OR teenage) AND (South Africa OR Gauteng OR Western Cape OR Mpumalanga OR Limpopo OR North-West OR Free-State OR Eastern Cape OR Northern Cape OR Kwazulu-Natal). Relevant articles published between 2000 and 2022 will be identified.

### 2.4. Article Screening

In the systematic review and meta-analysis protocol, the article screening process involves a multi-step approach. First, titles and abstracts are screened to identify relevant studies on tobacco use and behaviour among South African adolescents and young adults. Two reviewers will carefully scan and read the titles, abstracts, and keywords of all the articles retrieved from the databases to check the relevancy and eligibility. Population, Intervention (Exposure), Comparators, Outcomes, and Study design (PICOS) criteria will be used at the abstract level to outline the characteristics of studies to be selected for further review. Unrelated articles will be removed. Any discrepancies between the reviewers will be investigated and discussed, and where there are disagreements, the full texts will be reviewed. Furthermore, the full articles for the relevant abstracts will be checked and cross-checked for eligibility. At each stage, the reasons for study exclusion will be documented. This rigorous and methodical screening process ensures that the results of the review and meta-analysis are robust and reliable.

### 2.5. Primary Outcome and Determinants

The primary outcome of the study will be the prevalence of current tobacco smoking among adolescents and young adults in South Africa. The major determinants of smoking behaviour will include gender, race, household income, and level of education.

### 2.6. Inclusion and Exclusion Criteria

Studies will be included if they were conducted in South Africa or South African provinces (Gauteng, Western Cape, Mpumalanga, Limpopo, North-West, Free-State, Eastern Cape, Northern Cape, and Kwazulu-Natal), and if they include data on the prevalence of tobacco use among adolescents and young adults aged between 12 and 24 years. Moreover, studies will be selected if they are published in the peer-reviewed journals, conducted between 2000 and 2022, published in English language, and designed as cross-sectional and longitudinal studies.

Studies will be excluded if they were conducted outside South Africa, conducted before the year 2000, explored electronic cigarette use, were not full articles, published as part of books or conference proceedings, and were not peer-reviewed. Studies will also be excluded if they are qualitative, lacked quantitative details, or were designed as randomized clinical trials or systematic reviews.

### 2.7. Data Extraction

The data extraction will include the surname of the first author, the year the study was published, the study location and setting, design of the study, sampling technique, study population (general population, secondary school leaners, and university students), sample size, mean age of adolescents and young adults, prevalence of tobacco use, and gender. The reviewers will extract articles and save them in an Excel spreadsheet.

### 2.8. Risk of Bias Assessment and Quality Assessment

Risk of bias will be assessed qualitatively concerning selection bias (sample population), selection bias (participation rate), reporting bias (selective outcome reporting), performance bias (analytical methods for bias control), and other biases. Risk of bias analysis will conducted by rating studies using low, unclear, and high risk of bias using the Risk of Bias Assessment Tool for Non-Randomised Studies [28]. The Joanna Briggs Institute (JBI) checklist for studies reporting prevalence data [29] will be used to thoroughly evaluate the quality of the selected studies. To achieve these, 9 questions posed on the checklist will be explored. These questions are related to whether the (a) sample frame is appropriate to address the target population; (b) study participants are sampled in an appropriate way; (c) sample size is adequate; (d) study subjects and setting are described in detail; (e) data analysis is conducted with sufficient coverage of the identified sample; (f) valid methods are used for the identification of the condition; (g) condition is measured in a standard, reliable way for all participants; (h) appropriate statistical analysis is performed; and (i) whether the response rate is adequate, and, if not, whether it was managed appropriately. The four possible replies to these questions are “yes”, “no”, “unclear”, or “not applicable”.

### 2.9. Statistical Analysis

The extracted data will be reported in a descriptive table to show the study characteristics. The table will show the authors, year of study, location, study participants, article type, gender, level of education, sample size, number of males and females, mean age, and prevalence (%). The pooled prevalence of current tobacco use among South African adolescents and young adults will be analysed using STATA, version 17 [30]. The heterogeneity of prevalence estimates will be assessed using I^2^ statistic (DerSimonian-Laid approach). The high I^2^ statistic values will be to show that most of the variability in included studies is due to heterogeneity rather than chance [31]. The I^2^ statistic value across studies will be considered low if it is 25%, moderate if it is 50%, and high if it is 75%. Studies will be reported in different groups, such as the general population, males, and females. All the prevalence data will be reported with computed 95% confidence intervals (CIs). Values of *p* < 0.05 will be considered significantly heterogeneous. Sensitivity analyses will be performed by excluding one study at a time to determine any change before and after the analysis, and to see if any study influenced the significance of observed estimates. A funnel plot, in terms of asymmetry, and Egger’s linear regression test will be used to assess publication bias [32,33]. Meta-regression will be performed according to the year of study and the sample size to estimate the impact of current tobacco smoking prevalence.

### 2.10. Ethics and Dissemination

As it will be a systematic review, without the involvement of human beings, there will be no requirement for ethical approval. Findings will be disseminated widely through peer-reviewed publication and in various media, for example, conferences, congresses, or symposia.

## 3. Discussion

This systematic review and meta-analysis study will be conducted to estimate the pooled prevalence of tobacco use among South African adolescents and young adults. The estimates will be compared with results found in other African countries and globally. The association between tobacco use and socio-demographics will also be assessed. Additionally, there are a lack of empirical studies conducted to systematically review the prevalence of tobacco use in South Africa. This piece will reveal critical insights into the public health challenge posed by this issue. It will underscore the urgent need for comprehensive and targeted interventions to counteract the rising prevalence of tobacco use in this demographic group, which is associated with significant health risks. This review will provide nuanced understandings of the varied determinants of tobacco use, from socio-cultural factors to the influence of the tobacco industry’s marketing strategies. This knowledge can help to shape more effective and targeted tobacco control policies and interventions.

The meta-analysis approach will provide essential results which will be used in designing better-tailored interventions among young people in South Africa. The focus on South Africa offers the potential for these findings to inform global strategies, given the country’s diverse socio-economic and cultural makeup. While the efforts made by the South African government to control tobacco use have had some effect, the study findings will highlight the need for a more multi-faceted approach, encompassing not just regulatory efforts but also education, support services, and community initiatives. These results can be used to inform further policy development and can be a useful tool for other nations grappling with similar public health issues.

The implementation of effective policies to curb tobacco use and associated behaviour among South African adolescents and young adults is critical to the nation’s public health strategy. Comprehensive strategies could incorporate efforts such as raising the legal age for tobacco purchase, regulating e-cigarette usage, and implementing and enforcing marketing restrictions on tobacco products. The results of the systematic review and meta-analysis can guide future policy designs, enabling them to be more targeted and thus more effective. This can lead to a significant decrease in the initiation and prevalence of smoking, reduce health disparities among young adults, and ultimately improve the overall public health outcomes in South Africa. Moreover, the findings can also have implications for shaping global tobacco control strategies, given the transferability of successful interventions across different populations and cultural contexts.

Strengths and Limitations of This Study

This study will provide valuable insights for the evolution of further policy development and may serve as a useful reference for other nations dealing with similar public health concerns. The limitations of this study may include differences in study design, populations, measurements, and outcomes among the studies; this may lead to high variability in the results, potentially affecting the reliability of the meta-analysis findings. Moreover, some studies may have insufficient smoking determinants that could be analysed in this study. Non-English electronic databases will not be searched. This limitation may cause language bias. Studies with negative or null results are less likely to be published than those with positive results. 

## Data Availability

Not applicable.
